# Ipatasertib plus paclitaxel for *PIK3CA/AKT1/PTEN*-altered hormone receptor-positive HER2-negative advanced breast cancer: primary results from cohort B of the IPATunity130 randomized phase 3 trial

**DOI:** 10.1007/s10549-021-06450-x

**Published:** 2021-12-03

**Authors:** Nicholas Turner, Rebecca A. Dent, Joyce O’Shaughnessy, Sung-Bae Kim, Steven J. Isakoff, Carlos Barrios, Shigehira Saji, Igor Bondarenko, Zbigniew Nowecki, Qinshu Lian, Sarah-Jayne Reilly, Heather Hinton, Matthew J. Wongchenko, Bruno Kovic, Aruna Mani, Mafalda Oliveira

**Affiliations:** 1 Breast Unit, The Royal Marsden NHS Foundation Trust, Fulham Road, London, SW3 6JJ UK; 2 Breast Cancer Now Research Centre, The Institute of Cancer Research, London, UK; 3 Division of Medical Oncology, National Cancer Centre Singapore, Singapore, Singapore; 4 Department of Medical Oncology, Texas Oncology, Baylor University Medical Center, US Oncology, Dallas, TX USA; 5 Department of Oncology, Asan Medical Center, University of Ulsan College of Medicine, Seoul, South Korea; 6 Division of Hematology and Oncology, Massachusetts General Hospital, Boston, MA USA; 7 Latin American Cooperative Oncology Group, Oncology Research Service, Hospital São Lucas, PUCRS, Porto Alegre, RS Brazil; 8 Department of Medical Oncology, Fukushima Medical University Hospital, Fukushima, Japan; 9 Oncology and Medical Radiology Department, City Clinical Hospital No. 4, Dnipropetrovsk, Ukraine; 10 Oncology Centre, Instytut im. Marii-Sklodowskiej, Warsaw, Poland; 11 Biostatistics, Genentech, Inc, South San Francisco, CA USA; 12 Pharma Development, Roche Products Ltd, Welwyn Garden City, UK; 13 Product Development Safety, F. Hoffmann-La Roche Ltd, Basel, Switzerland; 14 Oncology Biomarker Development, Genentech, Inc, South San Francisco, CA USA; 15 Patient-Centered Outcomes Research, Product Development, Hoffmann-La Roche Limited, Mississauga, ON Canada; 16 Product Development Oncology, Genentech, Inc, South San Francisco, CA USA; 17 Medical Oncology Department, Vall d’Hebron University Hospital, Vall d’Hebron Institute of Oncology (VHIO), Barcelona, Spain

**Keywords:** PI3K/AKT, Hormone receptor positive, HER2 negative, Ipatasertib, First-line

## Abstract

**Purpose:**

PI3K/AKT pathway alterations are frequent in hormone receptor-positive (HR+) breast cancers. IPATunity130 Cohort B investigated ipatasertib–paclitaxel in PI3K pathway-mutant HR+ unresectable locally advanced/metastatic breast cancer (aBC).

**Methods:**

Cohort B of the randomized, double-blind, placebo-controlled, phase 3 IPATunity130 trial enrolled patients with HR+ HER2-negative *PIK3CA*/*AKT1/PTEN*-altered measurable aBC who were considered inappropriate for endocrine-based therapy (demonstrated insensitivity to endocrine therapy or visceral crisis) and were candidates for taxane monotherapy. Patients with prior chemotherapy for aBC or relapse < 1 year since (neo)adjuvant chemotherapy were ineligible. Patients were randomized 2:1 to ipatasertib (400 mg, days 1–21) or placebo, plus paclitaxel (80 mg/m^2^, days 1, 8, 15), every 28 days until disease progression or unacceptable toxicity. The primary endpoint was investigator-assessed progression-free survival (PFS).

**Results:**

Overall, 146 patients were randomized to ipatasertib–paclitaxel and 76 to placebo–paclitaxel. In both arms, median investigator-assessed PFS was 9.3 months (hazard ratio, 1.00, 95% CI 0.71–1.40) and the objective response rate was 47%. Median paclitaxel duration was 6.9 versus 8.8 months in the ipatasertib–paclitaxel versus placebo–paclitaxel arms, respectively; median ipatasertib/placebo duration was 8.0 versus 9.1 months, respectively. The most common grade ≥ 3 adverse events were diarrhea (12% with ipatasertib–paclitaxel vs 1% with placebo–paclitaxel), neutrophil count decreased (9% vs 7%), neutropenia (8% vs 9%), peripheral neuropathy (7% vs 3%), peripheral sensory neuropathy (3% vs 5%) and hypertension (1% vs 5%).

**Conclusion:**

Adding ipatasertib to paclitaxel did not improve efficacy in *PIK3CA/AKT1/PTEN*-altered HR+ HER2-negative aBC. The ipatasertib–paclitaxel safety profile was consistent with each agent’s known adverse effects.

*Trial registration* NCT03337724.

**Supplementary Information:**

The online version contains supplementary material available at 10.1007/s10549-021-06450-x.

## Introduction

The phosphoinositide 3-kinase (PI3K)/AKT pathway is frequently upregulated in cancer [1, 2]. Activation of AKT, the central node of the PI3K/AKT pathway, promotes cell survival, proliferation, metabolism and growth [1, 3], and is implicated in resistance to endocrine therapy [4]. *PIK3CA/AKT1/PTEN* alterations are frequently observed in breast cancer, including approximately 50% of patients with hormone receptor-positive (HR+) breast cancers, and contribute towards a negative prognosis and resistance to endocrine therapies [5–9].

Ipatasertib is a highly selective oral ATP-competitive small-molecule inhibitor of all three AKT isoforms [10]. Ipatasertib is being developed for the treatment of cancers in which PI3K/AKT pathway activation may be relevant for tumor growth or therapeutic resistance, and has demonstrated PI3K/AKT pathway inhibition in preclinical studies [10–12]. PTEN protein loss and *PTEN* or *PIK3CA* genetic alterations appeared to be associated with enhanced sensitivity to single-agent ipatasertib in cell lines and preclinical models [10, 13]. In a phase 1b study, the combination of ipatasertib and paclitaxel was well tolerated and showed radiographic responses in patients with advanced/metastatic breast cancer, including HR+ disease [14]. In the randomized, phase 2 LOTUS trial, the addition of ipatasertib to paclitaxel improved progression-free survival (PFS) compared with paclitaxel alone in metastatic triple-negative breast cancer (TNBC), especially in patients whose tumors harbored alterations in *PIK3CA*, *AKT1* and/or *PTEN* [15].

The phase 3 IPATunity130 trial included two independent randomized cohorts (Cohort A in TNBC and Cohort B in HR+ HER2-negative [HER2–] unresectable locally advanced or metastatic breast cancer [aBC]) evaluating ipatasertib plus paclitaxel combination therapy and a third single-arm signal-seeking cohort in patients with TNBC whose tumors did not have *PIK3CA/AKT1/PTEN* alterations (Cohort C) evaluating a triplet combination of ipatasertib, paclitaxel and atezolizumab. The two randomized cohorts are powered independently and designed to be analyzed separately. Here we report results from Cohort B, which evaluated ipatasertib in combination with paclitaxel for HR+ HER2– *PIK3CA*/*AKT1/PTEN*-altered aBC.

## Patients and methods

### Study design and participants

In Cohort B of the IPATunity130 (NCT03337724) randomized, double-blind, placebo-controlled, phase 3 trial, eligible patients had to have HR+ (≥ 1% staining) HER2– *PIK3CA/AKT1/PTEN*-altered measurable aBC according to Response Evaluation Criteria in Solid Tumors (RECIST; version 1.1). Tumor *PIK3CA/AKT1/PTEN* alteration status (i.e., activating alterations in *PIK3CA* and/or *AKT1*, and/or inactivating alterations in *PTEN*, described in detail in Supplementary Table S1) was determined from the most recently available tumor tissue sample using the Foundation Medicine Inc (Cambridge, MA) next-generation sequencing Clinical Trial Assay (CTA). In addition, patients had to be inappropriate for endocrine-based therapy (i.e., demonstrated insensitivity to endocrine therapy or visceral crisis), a candidate for taxane monotherapy and have Eastern Cooperative Oncology Group performance status 0 or 1. Patients who had previously received chemotherapy for aBC or whose diagnosis of aBC was < 1 year since their last (neo)adjuvant chemotherapy were ineligible, as were patients with a history of or known presence of brain or spinal cord metastases. Prior cyclin-dependent kinase (CDK)4/6 inhibitors and PI3K/mammalian target of rapamycin (mTOR) inhibitors were permitted.

### Procedures

Patients were randomized in a 2:1 ratio by investigators using an interactive web-response system to receive either oral ipatasertib (400 mg daily on days 1–21) plus intravenous paclitaxel (80 mg/m^2^ on days 1, 8 and 15) of a 28-day cycle, or placebo plus the same paclitaxel regimen. Randomization was stratified by three criteria: (neo)adjuvant chemotherapy (yes vs no), prior PI3K/mTOR inhibitor (yes vs no) and region (Asia–Pacific vs Europe vs North America vs rest of the world). To improve the management of diarrhea (commonly associated with ipatasertib and/or paclitaxel therapy), antidiarrheal prophylaxis (loperamide) was mandated for the first cycle for all patients, where permitted locally. Treatment was continued until disease progression (RECIST; version 1.1), unacceptable toxicity or patient withdrawal. Patients discontinuing paclitaxel or ipatasertib/placebo because of toxicity could continue on single-agent treatment. Crossover from placebo to ipatasertib was not permitted.

Tumors were assessed every 8 weeks by the investigators according to RECIST (version 1.1). After discontinuing treatment, patients were followed up every 3 months for survival and subsequent anticancer therapies. Patient-reported outcomes (PROs) were assessed using selected scales of the European Organisation for Research and Treatment of Cancer Quality of Life Questionnaire Core 30 (EORTC QLQ-C30), administered at baseline, at day 1 of each subsequent cycle, and at the treatment discontinuation visit. Adverse events (AEs) were assessed and graded according to Common Terminology Criteria for Adverse Events (version 4.0).

### Endpoints

The primary objective was to assess the efficacy of the ipatasertib plus paclitaxel combination as determined by investigator-assessed PFS. PFS was defined as the interval between randomization and the first occurrence of disease progression, as determined by the investigator according to RECIST (version 1.1), or death from any cause, whichever occurred first. A sensitivity analysis of PFS according to independent review committee (IRC) assessment was performed in a similar manner.

Overall survival (OS; defined as the interval between randomization and death from any cause) was the key secondary endpoint. Other secondary endpoints included confirmed objective response rate (investigator assessed per RECIST [version 1.1]), duration of response in responding patients, clinical benefit rate (complete or partial response, or stable disease sustained for ≥ 24 weeks) in patients with measurable disease at baseline, PROs, and safety.

### Statistical analysis

The planned sample size was 201 patients. For the primary analysis, 150 PFS events in the intent-to-treat (ITT) population were required to detect a hazard ratio of 0.62 with 80% power at a two-sided significance level of 5%. This corresponds to an increase in median PFS from 8.5 months in the control arm to 13.8 months in the ipatasertib-containing arm. If investigator-assessed PFS in the ITT population was significant at 5%, OS was to be tested hierarchically at the same significance level.

Efficacy analyses were based on all randomly assigned patients (ITT population) according to the treatment arm to which patients were allocated. PRO analyses of Global Health Status/Quality of Life (GHS/QoL) were performed on randomized patients who had a baseline and at least one post-baseline PRO assessment (PRO-evaluable population); PRO analyses of time to ≥ 11-point confirmed deterioration in pain [16] were performed on the ITT population. Safety analyses were based on all patients who received at least one dose of ipatasertib, placebo or paclitaxel; patients were analyzed based on the treatment actually received.

## Results

### Patient population

**Fig. 1:**
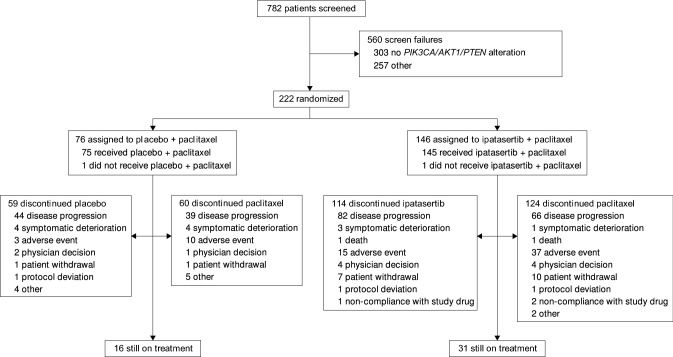
Patient profile

Between January 6, 2018 and March 29, 2019, 782 patients were screened for the trial, of whom 560 were considered screen failures, most commonly because of absence of *PIK3CA/AKT1/PTEN* alteration (*n* = 303). Ultimately, 222 patients were randomized: 146 to ipatasertib plus paclitaxel and 76 to placebo plus paclitaxel. Two patients in the ipatasertib plus paclitaxel arm were included in the efficacy analyses despite the most recent hormone receptor status identifying tumors as TNBC. Two patients (one in each arm) received no treatment and were therefore excluded from the safety analysis population (Fig. 1). The majority of samples for determination of *PIK3CA/AKT1/PTEN* status were from primary tumor tissue (143 [64%] primary, 66 [30%] metastatic, 10 [5%] unknown, three [1%] enrolled based on local testing with no central confirmation available).

Baseline characteristics were generally well balanced, except for a higher proportion of patients in the ipatasertib plus paclitaxel arm with a disease-free interval of > 3 years (40% vs 29% in the placebo plus paclitaxel arm) or a chemotherapy-free interval > 3 years (31% vs 24%, respectively) (Table 1). Prior therapy was balanced between arms and included (neo)adjuvant chemotherapy in 55% of patients, endocrine therapy for aBC in 46%, PI3K/mTOR inhibitor in 24% (predominantly everolimus) and CDK4/6 inhibitor in 26%. According to European Society for Medical Oncology (ESMO) definitions [17], 18% of patients had primary endocrine resistance and 45% had secondary endocrine resistance. A further 18% of patients did not meet the ESMO definitions for endocrine resistance but were deemed by the investigator to have visceral crisis. Within the subset of 120 patients who had received no prior endocrine therapy in the advanced setting, 18 (15%) had primary endocrine resistance, 34 (28%) had secondary endocrine resistance and 39 (33%) had visceral crisis without endocrine resistance.

**Table 1:** Baseline characteristics

Characteristic	Placebo + paclitaxel (*n* = 76)	Ipatasertib + paclitaxel (*n* = 146)
Median age, years (range)	56 (28–77)	57.5 (30–81)
Postmenopausal, *n* (%)	59 (78)	113 (78)^a^
Region, *n* (%)^b^		
Asia–Pacific	21 (28)	37 (25)
Europe	36 (47)	74 (51)
North America	6 (8)	7 (5)
Rest of world	13 (17)	28 (19)
Prior (neo)adjuvant chemotherapy, *n* (%)^b^	43 (57)	80 (55)
Prior PI3K/mTOR inhibitor, *n* (%)^b^	17 (22)	36 (25)
Prior CDK4/6 inhibitor use, *n* (%)	21 (28)	36 (25)
Disease-free interval, years, *n* (%)^c^		
< 1	3 (4)	5 (3)
1–3	21 (28)	29 (20)
> 3	22 (29)	58 (40)
No prior breast surgery	23 (30)	43 (29)
Not available	7 (9)	11 (8)
Chemotherapy-free interval, *n* (%)		
1–3 years	18 (24)	29 (20)
> 3 years	18 (24)	45 (31)
No prior chemotherapy	36 (47)	65 (45)
Not available	4 (5)	7 (5)
Metastatic disease at baseline, *n* (%)	74 (97)	141 (97)
Metastatic sites, *n* (%)^d^		
Lung	35 (46)	52 (36)
Liver	43 (57)	70 (48)
Bone	54 (71)	95 (65)
Lymph node	38 (50)	80 (55)
Visceral disease, *n* (%)	63 (83)	114 (78)
No. of lines of prior endocrine treatment in advanced setting, *n* (%)		
0	39 (51)	81 (55)
1	17 (22)	34 (23)
2	9 (12)	15 (10)
3 +	11 (14)	16 (11)
Endocrine resistance status, *n* (%)^e^		
Primary	14 (18)	26 (18)
Secondary	32 (42)	67 (46)
Visceral crisis without endocrine resistance	10 (13)	29 (20)
FMI status, *n* (%)^f^		
* PIK3CA/AKT1* alteration	60 (81)	127 (88)
* PTEN*-only alteration	14 (19)	17 (12)

^a^Not applicable in two male patients; ^b^As recorded in interactive web-response system; ^c^Defined as the interval between final breast surgery with curative intent and initial diagnosis of locally advanced/metastatic breast cancer; ^d^Missing in one patient in the placebo + paclitaxel arm; ^e^Categories are mutually exclusive; ^f^FMI mutation status missing in two patients in each arm

*CDK* cyclin-dependent kinase, *FMI* Foundation Medicine Inc, *PI3K* phosphoinositide 3-kinase

### Efficacy

**Fig. 2:**
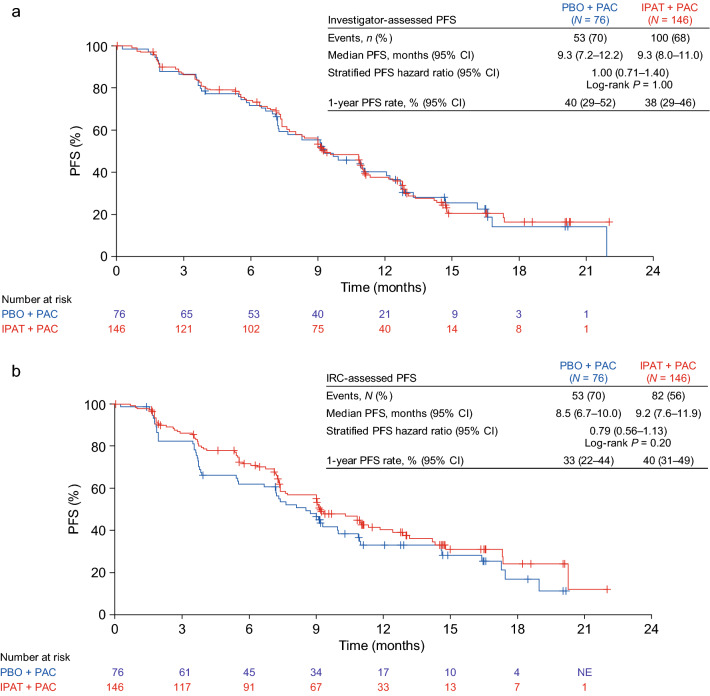
PFS. **a** investigator assessed; **b** IRC assessed. *IPAT* ipatasertib, *NE* not evaluable, *PAC* paclitaxel, *PBO* placebo

**Fig. 3:**
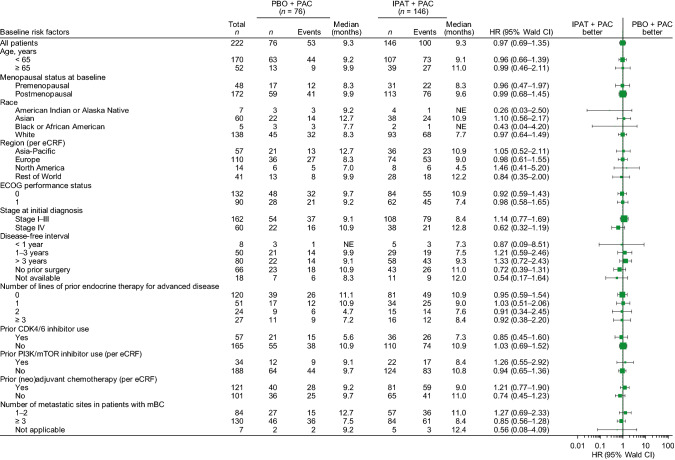
PFS in subgroups. Data for race ‘multiple’ (two patients in the placebo arm, none in the ipatasertib arm), race ‘unknown’ (one patient in the placebo arm without an event, nine patients in the ipatasertib arm) and number of metastatic sites in patients with mBC ‘0’ (one patient in the placebo arm without an event) are not shown because hazard ratios could not be calculated with events in only one arm. *ECOG* Eastern Cooperative Oncology Group, *eCRF* electronic case report form, *HR* hazard ratio, *IPAT* ipatasertib, *mBC* metastatic breast cancer, *NE* not evaluable, *PAC* paclitaxel, *PBO* placebo

At the clinical cutoff date (January 17, 2020), the median duration of follow-up in the overall population was 12.9 months (range 0–23.3 months) and was similar in the two treatment arms. Median investigator-assessed PFS was 9.3 months in both arms (95% confidence interval [CI] 8.0–11.0 months in the ipatasertib plus paclitaxel arm and 7.2–12.2 months in the placebo plus paclitaxel arm). The PFS hazard ratio was 1.00 (95% CI 0.71–1.40; log-rank *p* = 0.997) (Fig. 2a). The 1-year PFS rate was 38% (95% CI 29–46%) in the ipatasertib plus paclitaxel arm and 40% (95% CI 29–52%) in the placebo plus paclitaxel arm. IRC-assessed PFS results were consistent with investigator-assessed PFS (median 9.2 months [95% CI 7.6–11.9 months] with ipatasertib plus paclitaxel vs 8.5 months [95% CI 6.7–10.0 months] with placebo plus paclitaxel). The IRC-assessed PFS hazard ratio was 0.79 (95% CI 0.56–1.13; Fig. 2b). Subgroup analyses of PFS showed consistent results across all populations analyzed and no subgroup deriving a benefit from ipatasertib was identified (Fig. 3).

Among the 144 patients in the ipatasertib plus paclitaxel arm and 75 in the placebo plus paclitaxel arm with measurable disease, the objective response rate was 47% in both arms (95% CI 38–58% and 35–59%, respectively), including complete response in four patients (3%) in the ipatasertib plus paclitaxel arm versus none in the placebo plus paclitaxel arm. The median duration of response was 9.2 months in both arms (95% CI 7.2–11.3 months in the 67 responders in the ipatasertib plus paclitaxel arm; 95% CI 6.8–12.5 months in the 35 responders in the placebo plus paclitaxel arm). The clinical benefit rate was 69% (95% CI 61–76%) in the ipatasertib plus paclitaxel arm and 65% (95% CI 53–76%) in the placebo plus paclitaxel arm.

OS results were immature (deaths in 23% of the ipatasertib plus paclitaxel arm vs 29% of the placebo plus paclitaxel arm). At this interim analysis, median OS was not evaluable in ipatasertib-treated patients and 20.9 months (95% CI 17.3–not evaluable) in the placebo plus paclitaxel arm (hazard ratio, 0.72, 95% CI 0.42–1.24).

### Patient-reported outcomes

Completion rates for PRO questionnaires exceeded 80% in each arm up to cycle 23 and at the study drug discontinuation visit. Overall, 207 patients were evaluable for mean change from baseline in GHS/QoL (134 in the ipatasertib plus paclitaxel arm, 73 in the placebo plus paclitaxel arm).

Patients’ GHS/QoL mean scores at baseline were 68.8 in the ipatasertib plus paclitaxel arm and 63.7 in the placebo plus paclitaxel arm, and were maintained in both treatment arms until Cycle 10 (at which point, less than half of the PRO-evaluable population in each arm remained on treatment, precluding meaningful analysis beyond Cycle 10) (Supplementary Figure S1). No clinically meaningful deterioration (i.e., a ≥10-point decrease [18]) from baseline values was observed in either arm.

Median time to confirmed deterioration in pain (as measured by the pain scale of the EORTC QLQ-C30) was not evaluable in either treatment arm (confirmed deterioration in 38% of patients in the ipatasertib plus paclitaxel arm versus 30% in the placebo plus paclitaxel arm). However, the Kaplan–Meier plot of time to confirmed ≥11-point deterioration in pain from baseline showed a sustained separation of the curves at 6 months in favor of the placebo plus paclitaxel arm (hazard ratio, 1.36; 95% CI 0.83–2.22) (Supplementary Figure S2).

### Safety

At the clinical cutoff date, 31 patients (21%) randomized to ipatasertib plus paclitaxel and 16 (21%) randomized to placebo plus paclitaxel remained on treatment. Treatment exposure is shown in Table 2. Mean paclitaxel dose intensity was similar in the two treatment arms, but the median duration of paclitaxel exposure was longer in the placebo plus paclitaxel arm than the ipatasertib plus paclitaxel arm. The most common AEs of any grade in patients who received ipatasertib plus paclitaxel were diarrhea (85%; grade 3 in 12%, grade 2 in 43%, grade 1 in 30%), alopecia (50%) and nausea (41%) (Table 3). Rash was slightly more common in ipatasertib-treated patients and the incidence of hyperglycemia was similar in the two arms.

**Table 2:** Treatment exposure

Treatment exposure	Placebo + paclitaxel	Ipatasertib + paclitaxel
	(*n* = 75)	(*n* = 145)
Patients still on treatment, *n* (%)	16 (21)	31 (21)
Median (range) duration of treatment, months		
Ipatasertib/placebo	9.1 (0–22)	8.0 (0–22)
Paclitaxel	8.8 (0–22)	6.9 (0–22)
Mean (SD) dose intensity, *(*%)^a^		
Ipatasertib/placebo	98.8 (2.9)	95.5 (10.1)
Paclitaxel	98.1 (6.0)	97.5 (12.0)
Mean (SD) cumulative dose, mg		
Ipatasertib/placebo	NA	69 691 (48 348)
Paclitaxel	3727 (2031)	3313 (2104)
Patients with AE leading to treatment discontinuation, *n* (%)	10 (13)	44 (30)
Ipatasertib/placebo	3 (4)	16 (11)
Paclitaxel	10 (13)	38 (26)
Patients with AE leading to dose reduction, *n* (%)	20 (27)	67 (46)
Ipatasertib/placebo	6 (8)	50 (34)
Paclitaxel	18 (24)	38 (26)
Patients with dose interruption, *n* (%)	43 (57)	86 (59)
Ipatasertib/placebo	32 (43)	63 (43)
Paclitaxel	38 (51)	77 (53)

^a^With respect to total number of doses

*AE* adverse event, *SD* standard deviation

**Table 3:** Summary of most common adverse events (≥ 20% any grade, ≥ 5% grade ≥ 3 in either arm)

Adverse event, *n* (%)	Placebo + paclitaxel (*n* = 75)		Ipatasertib + paclitaxel (*n* = 145)	
	Any grade	Grade ≥ 3	Any grade	Grade ≥ 3
Diarrhea	28 (37)	1 (1)	123 (85)	17 (12)
Alopecia	44 (59)	0	72 (50)	0
Nausea	15 (20)	0	60 (41)	2 (1)
Neuropathy peripheral	12 (16)	2 (3)	46 (32)	10 (7)
Anemia	15 (20)	0	43 (30)	1 (1)
Vomiting	5 (7)	0	42 (29)	3 (2)
Constipation	23 (31)	0	39 (27)	0
Neutropenia	18 (24)	7 (9)	38 (26)	12 (8)
Rash	9 (12)	0	29 (20)	2 (1)
Fatigue	18 (24)	3 (4)	27 (19)	0
Asthenia	13 (17)	2 (3)	27 (19)	2 (1)
Peripheral sensory neuropathy	22 (29)	4 (5)	23 (16)	4 (3)
Neutrophil count decreased	17 (23)	5 (7)	23 (16)	13 (9)
Hyperglycemia	9 (12)	0	20 (14)	3 (2)
ALT increased	15 (20)	3 (4)	17 (12)	7 (5)
WBC decreased	5 (7)	1 (1)	10 (7)	4 (3)
Lipase increased	3 (4)	2 (3)	5 (3)	3 (2)
Hypertension	4 (5)	4 (5)	7 (5)	2 (1)

*ALT* alanine aminotransferase; *WBC* white blood cell

Grade ≥ 3 AEs occurred in 55% of patients in the ipatasertib plus paclitaxel arm and 47% in the placebo plus paclitaxel arm. The most common grade ≥ 3 AEs reported in either treatment group (ipatasertib plus paclitaxel vs placebo plus paclitaxel) were diarrhea (12% vs 1%, respectively [no grade 4 episodes]), neutrophil count decreased (9% vs 7%), neutropenia (8% vs 9%), peripheral neuropathy (7% vs 3%), peripheral sensory neuropathy (3% vs 5%) and hypertension (1% vs 5%). Incidences of selected AEs of specific relevance to ipatasertib are shown in Supplementary Table S2. AEs leading to ipatasertib/placebo discontinuation included diarrhea (3% vs 1%), febrile neutropenia (1% vs 0%) and hyperglycemia (1% vs 0%). The AEs most commonly leading to paclitaxel discontinuation were peripheral sensory neuropathy (5% in both arms), peripheral neuropathy (6% vs 1%), neutrophil count decreased (1% vs 3%) and febrile neutropenia (2% vs 0%).

Most patients (92% in the ipatasertib plus paclitaxel arm vs 82% in the placebo plus paclitaxel arm) received at least one dose of loperamide for diarrhea prophylaxis or treatment. The proportion receiving prophylactic loperamide was similar in the two treatment arms (61% vs 64%, respectively) but a higher proportion of patients in the ipatasertib plus paclitaxel arm received loperamide to treat diarrhea (78% vs 29%, respectively). In the ipatasertib plus paclitaxel arm, 96% of diarrhea episodes (431 of 448) resolved. The median time to resolution of the first episode of diarrhea (any grade) was 15 days (95% CI 8–18 days) and the median duration of the first episode of grade ≥ 3 diarrhea was 3 days (95% CI 1–6 days).

Serious AEs were more common in the ipatasertib plus paclitaxel arm (19%) than in the placebo plus paclitaxel arm (12%). AEs were fatal in five patients (3%) in the ipatasertib plus paclitaxel arm and one patient (1%) in the placebo plus paclitaxel arm; two of these deaths were considered related to study treatment (grade 5 febrile neutropenia related to both drugs in the ipatasertib plus paclitaxel arm and grade 5 sepsis related to paclitaxel in the placebo plus paclitaxel arm; both patients had visceral crisis at screening). The remaining four deaths in the ipatasertib plus paclitaxel arm were from hospital-acquired pneumonia, respiratory distress, unexplained death, and general physical health deterioration/road traffic accident (each reported in one patient).

## Discussion

In Cohort B of the randomized phase 3 IPATunity130 trial, adding ipatasertib to paclitaxel did not improve PFS in *PIK3CA/AKT1/PTEN*-altered HR+ HER2– aBC. Ipatasertib plus paclitaxel was well tolerated, and the safety profile of the regimen was consistent with the known risks of each agent. No new safety signals were identified. OS follow-up is ongoing.

The results from IPATunity130 Cohort B are consistent with findings from the randomized, phase 2 BEECH trial of the oral AKT inhibitor capivasertib in combination with first-line paclitaxel in HR+ HER– aBC [19]. In BEECH, similar to the present trial, combining an AKT inhibitor with paclitaxel did not significantly improve PFS in either the overall population or the *PIK3CA*-altered population. The target patient population for IPATunity130 Cohort B was patients with endocrine-resistant disease; however, only a quarter of patients had received prior CDK4/6 inhibitors. Although it is tempting to hypothesize that enrollment of a less endocrine-resistant patient population could explain the lack of PFS benefit, subgroup analyses do not support this hypothesis. The subgroup of patients with greater exposure to prior endocrine therapy did not show enhanced benefit from ipatasertib (Fig. 3).

Another possible explanation for the lack of benefit is the higher proportion of patients discontinuing paclitaxel because of AEs in the ipatasertib arm, which may have compromised the efficacy of paclitaxel. Patients in the ipatasertib plus paclitaxel arm received a shorter duration and lower cumulative dose of paclitaxel than those in the placebo plus paclitaxel arm. This may have limited the ability to isolate the effect of ipatasertib. Of note, median PFS was identical in the two treatment arms and there was no signal of benefit from ipatasertib. There may be important lessons to learn from the duration and intensity of paclitaxel exposure, and the challenges of introducing a drug in HR+ HER2– breast cancer with side effects differing from those of endocrine therapies. Of note, a similar proportion of patients (approximately one-third) in each arm experienced a confirmed deterioration in pain, and patients’ baseline quality of life was maintained while receiving ipatasertib plus paclitaxel treatment, showing no detrimental effect on patients’ overall quality of life with ipatasertib.

Consistent with the safety profile of ipatasertib plus paclitaxel observed in the LOTUS trial [15], there was more all-grade diarrhea, nausea and vomiting with ipatasertib. The incidence of diarrhea was lower in IPATunity130 Cohort B than in LOTUS, with only half as many ipatasertib-treated patients experiencing grade 3 diarrhea (11% in IPATunity130 Cohort B vs 23% in LOTUS). The observed reduction may be explained by the implementation of several diarrhea management measures in the IPATunity130 trial design, including prophylactic loperamide administration, improved patient education and AE management guidance, as well as greater investigator awareness and familiarity with the drug.

Hyperglycemia has been observed in various clinical trials of drugs targeting the PI3K/AKT pathway [20–22] and is generally considered to be a class effect of these therapies. However, in IPATunity130 Cohort B, the proportion of patients experiencing hyperglycemia was lower than in trials of other PI3K/AKT inhibitors [20, 23–25] and similar in the two treatment arms (14% with ipatasertib plus paclitaxel vs 12% with placebo plus paclitaxel). The proportion of patients with grade ≥ 3 hyperglycemia was low (2% vs 0%, respectively).

Overall, results from IPATunity130 Cohort B and the BEECH [19] trial differ from findings of trials combining a PI3K/AKT inhibitor with endocrine therapy (SOLAR-1 [20] and FAKTION [26]). PI3K/AKT signaling promotes estrogen-independent growth of HR+ HER2– breast cancer cells, which can be inhibited by combining PI3K inhibitors with anti-estrogens [8, 9, 27–29]. The SOLAR-1 randomized, phase 3 trial combined the PI3K inhibitor alpelisib with fulvestrant in patients with *PIK3CA*-mutant HR+ HER2– breast cancer [20] and the FAKTION trial combined the oral AKT inhibitor capivasertib with fulvestrant after relapse or progression on an aromatase inhibitor [26]. In line with preclinical findings, both of the trials showed a PFS benefit from the addition of a PI3K/AKT pathway inhibitor to endocrine therapy.

Considering all available data for AKT inhibition in HR+ HER2– aBC, it appears that endocrine blockade may be essential for efficacy in this setting. AKT induces endocrine receptor signaling, which may counter the potential benefit of an AKT inhibitor. Taken together, these results suggest that the benefit of AKT inhibition will be greatest if estrogen receptors are targeted alongside AKT inhibition. Ongoing trials of ipatasertib in breast cancer focus on combinations with endocrine therapy and/or immunotherapy.

## Supplementary Information

Below is the link to the electronic supplementary material.

Supplementary file1 (DOCX 363 kb)

Supplementary file2 (PDF 175 kb)

## Data Availability

Qualified researchers may request access to individual patient-level data through the clinical study data request platform (https://vivli.org/). Further details on Roche’s criteria for eligible studies are available here (https://vivli.org/members/ourmembers/). For further details on Roche’s Global Policy on the Sharing of Clinical Information and how to request access to related clinical study documents, see here (https://www.roche.com/research_and_development/who_we_are_how_we_work/clinical_trials/our_commitment_to_data_sharing.htm).

## Abbreviations

aBC: Advanced or metastatic breast cancer
AE: Adverse event
CDK: Cyclin-dependent kinase
CI: Confidence interval
CTA: Clinical Trial Assay
EORTC: European Organisation for Research and Treatment of Cancer
GHS/QoL: Global Health Status/Quality of Life
HER–: HER2-negative
HR+: Hormone receptor-positive
IRC: Independent review committee
ITT: Intent-to-treat
mTOR: Mammalian target of rapamycin
OS: Overall survival
PFS: Progression-free survival
PI3K: Phosphoinositide 3-kinase
PROs: Patient-reported outcomes
QLQ-C30: Quality of Life Questionnaire Core 30
RECIST: Response Evaluation Criteria in Solid Tumors
TNBC: Triple-negative breast cancer
