# Supplementary material for: Ipatasertib plus paclitaxel for PIK3CA/AKT1/PTEN-altered hormone receptor-positive HER2-negative advanced breast cancer: primary results from cohort B of the IPATunity130 randomized phase 3 trial
Source: Breast Cancer Res Treat. 2021 Dec 3;191(3):565–76. doi: 10.1007/s10549-021-06450-x (PMC8831286; doi:10.1007/s10549-021-06450-x)
Supplement: Supplementary file 1 — Supplementary file1 (DOCX 363 kb) [file 10549_2021_6450_MOESM1_ESM.docx]

**Supplementary Figure S1. Mean change from baseline in GHS/QoL (EORTC QLQ-C30) over time (PRO-evaluable population)**

*CI* confidence interval, *IPAT* ipatasertib, *PAC* paclitaxel, *PBO* placebo

**Supplementary Figure S2. Time to confirmed ≥11-point deterioration in pain over time (ITT population)**

*IPAT* ipatasertib, *NE* not evaluable, *PAC* paclitaxel, *PBO* placebo, *TTD* time to deterioration
