# Supplementary material for: Ipatasertib plus paclitaxel for PIK3CA/AKT1/PTEN-altered hormone receptor-positive HER2-negative advanced breast cancer: primary results from cohort B of the IPATunity130 randomized phase 3 trial
Source: Breast Cancer Res Treat. 2021 Dec 3;191(3):565–76. doi: 10.1007/s10549-021-06450-x (PMC8831286; doi:10.1007/s10549-021-06450-x)
Supplement: Supplementary file 2 — Supplementary file2 (PDF 175 kb) [file 10549_2021_6450_MOESM2_ESM.pdf]

Supplementary tables

Ipatasertib plus paclitaxel for *PIK3CA/AKT1/PTEN*-altered hormone receptor-positive HER2-negative advanced breast cancer: primary results from cohort B of the IPATunity130 randomized, phase 3 trial

Nicholas Turner[1,2] · Rebecca A. Dent[3] · Joyce O'Shaughnessy[4] · Sung-Bae Kim[5] · Steven J. Isakoff[6] · Carlos Barrios[7] · Shigehira Saji[8] · Igor Bondarenko[9] · Zbigniew Nowecki[10] · Qinshu Lian[11] · Sarah-Jayne Reilly[12] · Heather Hinton[13] · Matthew J. Wongchenko[14] · Aruna Mani[15] · Mafalda Oliveira[16]

Corresponding author: Nicholas Turner, Breast Unit, The Royal Marsden NHS Foundation Trust, Fulham Road, London SW3 6JJ, UK. Phone: 44 207 352 8133; E-mail: Nick.Turner@icr.ac.uk

Journal: Breast Cancer Research and Treatment

Supplementary Table S1. Definition of *PIK3CA/AKT1/PTEN*-altered status

PIK3CA/AKT1/PTEN altered defined as the presence of one or more of the following:

AKT1 missense mutations that result in amino acid substitution at the following Residues: E17, L52 or Q79

PIK3CA missense mutations that result in amino acid substitution at the following Residues: R88, G106, K111, G118, N345, C420, E453, E542, E545, Q546, M1043, H1047 or G1049

PTEN alterations that meet any of the following criteria:

- Homozygous deletion (copy number of 0)
- Dominant negative short variant (eg, C124S, G129E, R130X)
- Loss of heterozygosity (LOH) with copy number of 1 without concomitant single-nucleotide variants
- One deleterious short variant (including insertions and deletions; classification criteria provided below) with a concomitant loss of the non-mutant *PTEN* allele defined by LOH with copy number of 1 or LOH with copy number ≥ 1:
    - Any protein-truncating mutations, including nonsense mutations and frameshift insertions and deletions
    - Any mutations in the consensus splice donor and acceptor sequence that disrupts the consensus, including insertions and deletions
    - Any missense or non-frameshift mutation that has been confirmed somatic as described in the COSMIC database
    - If there are two or more deleterious short variants under LOH, the patient will not be eligible for the study

Supplementary Table S2. Summary of selected adverse events (safety population)

Adverse event, *n* (%)	Placebo + paclitaxel (*n* = 75)		Ipatasertib + paclitaxel (*n* = 145)	
	Any grade	Grade ≥ 3	Any grade	Grade ≥ 3
Diarrhea	28 (37)	1 (1)	123 (85)	17 (12)
Peripheral neuropathy[a]	48 (64)	6 (8)	93 (64)	15 (10)
Neutropenia[b]	33 (44)	11 (15)	59 (41)	26 (18)
Asthenia/fatigue	30 (40)	4 (5)	53 (37)	2 (1)
Nausea	15 (20)	0	60 (41)	2 (1)
Erythropenia[c]	16 (21)	0	44 (30)	1 (1)
Rash[d]	15 (20)	0	44 (30)	5 (3)
Vomiting	5 (7)	0	42 (29)	3 (2)
Hepatotoxicity[e]	21 (28)	3 (4)	22 (15)	9 (6)
Oral mucositis[f]	9 (12)	0	27 (19)	0
Hyperglycemia[g]	9 (12)	0	23 (16)	3 (2)
Hyperlipidemia[h]	7 (9)	1 (1)	15 (10)	1 (1)
Pneumonia	3 (4)	1 (1)	5 (3)	1 (1)
Pneumonitis	0	0	6 (4)	1 (1)
Thrombocytopenia[i]	1 (1)	0	3 (2)	1 (1)
Erythema multiforme	0	0	1 (1)	1 (1)
Colitis[j]	0	0	2 (1)	1 (1)

[a]Includes neuropathy peripheral, peripheral sensory neuropathy, paresthesia, polyneuropathy, muscular weakness, neurotoxicity, hypoesthesia, peripheral motor neuropathy, dysesthesia, gait disturbance, hypotonia, neuritis; [b]Includes neutropenia, neutrophil count decreased, febrile neutropenia; [c]Includes anemia, hemoglobin decreased, hematocrit decreased; [d]Includes rash, rash maculo-papular, erythema, dermatitis, drug eruption, folliculitis, dermatitis allergic, dermatitis bullous, rash erythematous, rash papular, rash pruritic; [e]Includes alanine aminotransferase increased, aspartate aminotransferase

increased, blood alkaline phosphatase increased, hyperbilirubinemia, gamma-glutamyl transferase increased, blood bilirubin increased, cholestasis, hepatic function abnormal, hepatocellular injury, transaminases increased; [f]Includes Stomatitis, mucosal inflammation, mouth ulceration; [g]Includes hyperglycemia, blood glucose increased, diabetes mellitus, glycosylated hemoglobin increased, type 2 diabetes mellitus; [h]Includes blood cholesterol increased, hypertriglyceridemia, hypercholesterolemia, blood triglycerides increased, hyperlipidemia; [i]Includes thrombocytopenia, platelet count decreased; [j]Includes enterocolitis
